# Medical News and Miscellany

**Published:** 1892-04

**Authors:** 


### Medical News and Miscellany.

**Attention** is called to the new advertisement of the Three Chlorides, Renz & Henry, and also to that of the "Od Chemical Company."

**Dr. M. A. Saylor**, late of Gonzales county, Texas, has gone to Chicago for a six months stay in attendance upon the clinics of that great medical center.

**The Able Prof. Amick** having made such a bad break will hardly be able, we fear, to make an Amic(k)able adjustment of his relations with his colleagues.

**Dr. W. L. Barker**, the new Superintendent of the (new) S. W. Texas Insane Asylum at San Antonio, got through the Senate by a very close vote. His appointment was not confirmed till the last day of the extra session.

**McClelland's Regional Anatomy**, published by J. B. Lippincott Co.—The first volume of this work has been most critically reviewed in most of the leading journals of this country as well as several of the journals of Great Britian, and many of these reviews have been written by men eminent in the profession, and but one adverse notice has come under our observation. They all with one accord speak of the book in the very highest praise.

**In Leucorrhœa or "Whites"** a most distressing and very common affection, especially amongst the leisure classes, injections of Peroxide of Hydrogen will give prompt relief by checking the discharge and soothing the irritation. By means of a common glass or hard rubber syringe, copious injections should be administered twice or oftener a day of a mixture of one to three ounces of Marchand's Peroxide of Hydrogen (medicinal) to a pint of lukewarm water, according to the tenderness of the infected surface. This will be found very efficacious, and will speedily effect a cure in many cases.

**Endorsed.**—A letter from a prominent medical gentleman of Cincinnati—one of the best known men, perhaps, in the profession, and a colleague of Amick's in the medical college, says that the editorial in the March number of the JOURNAL on the "Latest Cure for Consumption" has been very generally handed around amongst physicians in Cincinnati and is universally endorsed; and that it will be reproduced there. "It will have an important influence in giving to Amick the status which quackery should always have in the medical profession," says our correspondent.

---

**To the Members of the State Association.**—Sample copies of this issue will be pretty generally distributed at the Tyler meeting. Those who have heretofore not given us their support are invited to carefully examine the JOURNAL, and to lend us their aid in the laudable work in which, for seven years, we have been engaged, of warring on quackery in and out of the profession, and demanding a conformity to the code by all who profess to be and call themselves physicians. We think the JOURNAL has claims on the better element of the profession.

---

**Scabies.**—*Creolin in.*—Dr. De Lallis reports the use of creolin in scabies in the form of a five per cent. ointment, rubbing it once daily into the affected parts. Only four such applications are said to be necessary to produce perfect cure. Creolin, in his opinion, is preferable to any other remedy for this purpose, especially possessing the advantage over sulphur of not producing any eczema of the skin, and not staining either the skin or the linen.—*Therapeutic Gazette.*

---

**Seborrhœa.**—*Ointment for.*—From the St. Louis *Courier of Medicine* for December, we quote a formula of Dr. W. A. Hardaway's, much used at his dermatological clinic: Salicylic acid, 1 scruple; percipitated sulphur, 1 drachm; vaseline, 1 ounce; oil of roses, q. s. Mix thoroughly. This unguent has a wide range of application, as, for example, in the diseases causing dandruff, and in tinea versicolor, keratosis senilis, and lupus erythematosus.—*New York Medical Jonrnal.*

---

**The New York Medical Journal** in its issue of August 22nd, 1891, contains another letter on this subject from Dr. J. A. Wessinger, of Howell, Mich., who answers the question in the affirmative, and says further:

"Dispensing one's own medicine is a sure protection against the cupidity and (often) incompetence of the druggist. I am free to say that all druggists are not alike in this matter, but from personal experience I *know* some of them do practice the grossest violations of honor in the case of a physician's prescription. During the past three years I have dispensed my own medicines, and shall continue to do so."—*The Alkaloid.*

---

The committee appointed at the last meeting of the American Medical Association to consider the best means for promoting the prosperity of the sections of the Association will hold an adjourned meeting in the Hotel Cadillæ, Detroit, Mich., June 6, at 3 p. m.

Members of the committee are requested to notify the chairman of their intention to be present at this meeting.

The committee would esteem it a favor if each member of the Association would communicate in writing his or her views concerning the best measures for promoting the development of the sections. Such communications may be sent to the chairman of committee, John S. Marshall, M. D., 9 Jackson St., Chicago.

---

**Prof. J. E. Thompson**, of the Texas Medical College, Galveston, is at this writing (April 12), we regret to say, quite sick with fever at the Driskill Hotel, Austin. When taken sick some two weeks ago he came up to Austin in the hope that a change from the humid atmosphere of Galveston to the pure, dry air at Austin, would be beneficial. He placed himself in the care of our veteran physician Dr. W. A. Morris, and we are gratified to be able to say, is improving, with some hope of being able to be present at the College Commencement on the 22nd inst. It will be remembered that Prof. Seth M. Morris, son of Dr. W. A. Morris, was taken sick in Galveston in March, and that his father brought him to Austin to be under his care. Dr. Morris got well rapidly and returned to his post of duty on the 2nd inst.

---

**Postponed.**—It was announced in our March issue that this, April, number would be a memorial edition, devoted largely to a history of the Medical Department of the University of Texas, and biographical sketches of the Faculty; to be adorned with portraits of the eight Professors and cuts of the college building and Seely Hospital. We regret to say that owing to the delay in getting the photographs we are unable to get the engravings in time for this issue. They are now in the hands of the engraver, and the write-up will either appear in the May number of the JOURNAL, or we may postpone it to July, the beginning of our eighth year, when a double edition (2000) copies will be printed, and on better paper—the size of the JOURNAL being slightly enlarged. This will be a very expensive number, and we solicit, in advance, orders for extra copies, and new subscriptions.

---

Pediculosis Pubis.—In a clinical lecture (*L' Union Médicale*, February 26, 1891) on pediculosis pubis, Fournier very properly decries the treatment of this condition with blue ointment, inasmuch as this method is not only dirty and otherwise offensive, but it commonly gives rise the so-called eczema mercuriale, and may also, through absorption, bring about moderate or severe ptyalism. Much preferable and equally efficacious are an ointment of calomel, 1 to 20; baths of corrosive sublimate; a lotion consisting of 400 parts of water, 100 parts of alcohol, and 1 part of corrosive sublimate; and one consisting of 300 parts of vinegar, and 1 part of corrosive sublimate, to be applied diluted with 1 or 2 parts of water. As to the removal of the ova, the same method is to be practised as in pediculosis capitis—that is, with washings of warm vinegar, slightly diluted, and subsequent combings.

---

The Philadelphia Polyclinic.—This enterprising college for physicians is forging rapidly to the forefront of similar institutions in America. Under a new management more enterprise has been infused into it. Equipped with every facility for imparting information and demonstrating and illustrating every phase of the most advanced practice in every branch of medicine, in the hands of experienced and able clinicians, the school justly ranks with the best in the world. One advantage especially, will be appreciated by Texas physicians: the class is never allowed to get so large as to be unwieldy, and every individual matriculant receives personal and especial attention; every facility is given him to see and hear each clinic, and to make himself familiar with the tecnique in all operations.

The Polyclinic building is the handsomest in America, and was occupied for the first time last spring. Clinical material is abundant, and teaching is carried on every hour every day. Many of the teachers are famous specialists whose works are authority; and, moreover,—the Faculty—who constitute the Staff of the Hospitals, of course have access, with their classes to all the general and special wards, at all hours. See advertisement and write to Dr. J. M. Baldy, the Dean, for catalogue, mentioning the JOURNAL.

### MINUTE ON THE DEATH OF DR. D. HAYES AGNEW.

Adopted by the College of Physicians of Philadelphia, March 24, 1882.

The death of Dr. D. Hayes Agnew, recently President of the College, in the seventy-fourth year of his age, and after a life crowned with honor and usefulness, calls for an expression of the sense entertained by the College, of the gravity of the loss which it suffers, in common with the profession he adorned, the charitable institutions he served, and the community in which his skill did so mnch to lessen suffering and death.

He began his professional life with no adventitious aids; yet by incessant industry, indomitable perseverance, and singleness of purpose he attained to its highest rank. No temptation distracted his attention from the goal of his life; neither extraneous science, nor general literature, nor the allurements of art, nor the pleasures of society.

The undivided strength of his mind and his affections were devoted to enlarging the domain of surgery, not only in its operative methods, which he always subordinated to the welfare of his patients, but also in preparing for his profession a literary monument that might speak for him when his voice should be no longer heard.

His minute acquintance with anatomy, and his ambidextrous skill, enabled him to perform, with ease to himself and safety to his patients, operations which less accomplished surgeons hesitated to undertake.

He possessed a certain magnetism of manner, quite independent of formality, that evidently proceeded from the heart, and drew all hearts to himself. Never frivolous, but always cheerful, he was dignified, grave and earnest, making all who heard him as a teacher and speaker, or in familiar intercourse, recognize in him, above all other things, the upright man. For he possessed eloquence of conviction and the force of absolute honesty in all his statements, and thereby drew to himself as enthusiastic admirers and disciples, the successive classes of students whom he taught.

The College, desiring to show respect for the purity, uprightness, unselfishness and modesty of Dr. Agnew's character; its admiration for the noble example of his life; and its sense of the value of his contributions to the science and art of surgery, directs that this minute shall be duly recorded, and a copy of it, signed by the president and secretary, be conveyed to Dr. Agnew's family. Also, that the College will attend the funeral in a body, and that the presideut be requested to appoint a fellow to prepare a memoir of our late colleague.

CHARLES W. DULLES, M. D., Secretary.

### Epistaxis: An Easy and Effectual Method of Plugging.

Dr. A. A. Philip writes in the *Lancet:* Uudoubtedly, plugging the nares by aid of Bellocq's cannula is an excellent method; but occasionally, especially in country practice, Bellocq's cannula is not at hand, and some method easy, effectual, and effected by material always within reach, must be resorted to. Such a method I have found in the following: A piece of old, soft, thin cotton or silk, or oiled silk, about six inches square (a piece of an old handkerchief will answer) is taken, and, by means of a probe, metal thermometer case, or pen-holder, or anything handy, is pushed center first, "umbrella" fashion, into the nostril, the direction of pressure when the patient is sitting erect being backwards and slightly downwards. It is pushed on in this fashion until it is felt that the point of the "umbrella" is well into the cavity of the naso-pharynx. The thermometer case or probe, or whatever has been employed, is now pushed on in an upward direction, and then towards the sides, so as to pull more of the "umbrella" into the naso-pharynx. The thermometer case is now withdrawn. We have now a sac lying in the nares, its closed end protruding well into the pharynx behind, and its open and protruding at the anterior opening of the nares. If it be thought necessary, and is convenient, the inside of the sac may be brushed with some household astringeuts, such as alum solution, turpentine, etc. A considerable quantity of cotton-wool is now, by means of the thermometer case, pushed well back to the bottom of the sac. Then the thermometer case, being held firmly against the packed wool, the mouth of the sac is pulled upon, and thus its bottom with the wool packed in it is pulled forward, and forms a firm, hard plng wedged in the posterior nares. We may now pack the sac full of cotton-wool, dry or soaked in some astringent solution. The mouth of the sac may now be closed by tying it just outside the nostril with a piece of strong thread; it is then trimmed by scissors, and the ends of the thread secured outside.

The above method is easier than any I know, when both nostrils have to be plugged. It might be suggested to oil the cotton or silk in order to render its introduction easy, and prevent it adhering to the mucous membrane, and to render it easy of removal; but I have never found any difficulty without the oil, as the blood renders the material wet, and easy of introduction, while the oil does not facilitate removal, and may modify the effect of the astringents that may be used. The plug remains *in situ* as long as any other nose plug. In removing the plug, open the mouth of the sac, and with small dressing forceps remove the cotton-wool bit by bit; if there is bleeeding, simply syringe the sac with weak carbolic lotion or Condy's fluid, and repack with clean cotton-wool, or wool impregnated with some antiseptic. If there is no bleeding when the wool is picked out, gently pull out the sac; or if it be adhering to the mucous membrane, syringe in a little warm water, and it may then easily be removed. This method has many advantages. (*a*) It is easy, quickly accomplished, and effectual, and the materials are to be found in every house, and, indeed, about everybody's person (I have plugged in this manner, simply using a handkerchief, one part of which I used for the sac, and the other, torn into narrow strips, in place of the cotton-wool); (*b*) no damage is done to the floor of the nose, or back of the soft palate by strings, etc.; (*c*) no disagreeable hawking, coughing or vomiting takes place while the plug is introduced; (*d*) there are no disagreeable strings left hanging down the throat, causing coughing or sickness while the plug is in; (*e*) the plug can be removed gently without any force, so that no damage is done to the mucous membrane, and no return of hemorrhage caused. I employed this method frequently when in country practice, and do so now in bleeding after operation on the nares, and have always found it to be satisfactory. As the method has been of great use to me, and as I am not aware that any one has spoken of it before, I take this opportunity of mentioning it, in the hope that it may be of use to some brother practitioner when confronted by an urgent case of epistaxis, and other means of plugging are not at hand.—*Medical and Surgical Reporter.*

---

INDEX-CATALOGUE of the Library of the Surgeon-General's Office, U. S. Army. Authors and Subjects, vol. xii. Reger-Shuttleworth. Washington, D. C.: Government Printing Office, 1891. 1004 pages.

